# Effects of the Incidence Density of Fever (IDF) on Patients Resuscitated From In-Hospital Cardiac Arrest: A Mediation Analysis

**DOI:** 10.3389/fmed.2020.00086

**Published:** 2020-03-25

**Authors:** Yue Hu, Yong Guo, Xintao Wang, Yi Li, Dawei Sun, Derong Cui

**Affiliations:** ^1^Department of Anesthesiology, Shanghai Jiaotong University Affiliated Sixth People's Hospital, Shanghai, China; ^2^Department of Critical Care Medicine, Shanghai Jiaotong University Affiliated Sixth People's Hospital, Shanghai, China

**Keywords:** fever, cardiac arrest, survival rate, mediator, causal mediation analysis

## Abstract

**Objective:** The aim of this research was to study the factors contributing to the survival rate of in-hospital cardiac arrest (IHCA) and to determine whether the incidence density of fever (IDF) acts as a mediator.

**Methods:** Data from patients with IHCA who survived more than 48 h were collected from 2011 to 2017. IDF was defined as the fever duration divided by the hospitalization duration, prolonged fever was defined as fever lasting for more than 5 days, and early fever was defined as an initial onset within the first 2 days of IHCA. Possible clinical variables associated with IDF were examined by linear regression, and possible clinical variables associated with survival rate were examined by univariate and multivariate analyses. IDF was investigated as a mediator of the indirect effects of the risk factors on survival.

**Results:** In our retrospective study, the median IDF was 0, with an interquartile range from 0 to 0.42. Prolonged fever was noted in 16% (97/605) of the total, and early fever was noted in 17.2% (104/605) of the total. Linear regression results showed that positive chest X-ray, central venous catheter and Glasgow Coma Score (GCS) ≤ 8 were related to IDF. The IDF (OR: 0.36, 95% CI, 0.13–0.97, *P* = 0.04), prolonged fever (adjusted OR = 0.13, 95% CI, 0.06–0.29, *P* < 0.001), positive chest X-ray (OR: 0.67, 95% CI, 0.46–0.98, *P* = 0.04), central venous catheter placement (OR: 0.54, 95% CI, 0.34–0.89, *P* = 0.01), and endotracheal intubation (OR: 0.47, 95% CI, 0.33–0.69, *P* < 0.001) were also related to the negative outcome of hospital discharge after adjustment. Additionally, positive chest X-ray had a 19% effect on survival outcome through IDF as a mediator, and the indirect effect of central venous catheter mediated by IDF accounted for 10% of the total.

**Conclusions:** A higher IDF, prolonged fever, a positive chest X-ray, the use of a central venous catheter and endotracheal intubation reduced the survival rate of these patients, and the detrimental impacts of a positive chest X-ray and the use of a central venous catheter on survival outcomes were partially mediated by IDF.

## Introduction

In-hospital cardiac arrest (IHCA) is the leading cause of morbidity and mortality worldwide. Approximately 290,000 patients experience IHCA each year in the United States (1), and the survival rate ranges from 3.4 to 22.0% (2). Despite the return of spontaneous circulation (ROSC) after cardiopulmonary resuscitation, those who experience IHCA are still vulnerable to mortality and morbidity. Fortunately, timely and effective postresuscitation care can improve the survival rate among these weak patients (3).

Thus far, one of the primary challenges facing postresuscitation care is fever control. Fever, also known as hyperthermia, has a high incidence among patients resuscitated from IHCA (4). Moreover, both clinical and experimental evidence have demonstrated that fever in the early stage after ROSC is independently associated with increased morbidity and mortality due to IHCA (5–10). However, the treatment of IHCA patients with fever is very difficult. Fever control via the administration of antipyretic drugs or hypothermia therapy can promote significant harmful side effects, and the efficiency of these methods can be poor (11, 12). Nevertheless, clinical symptoms prior to the occurrence of fever are common, and early intervention can prevent the progression of fever (13).

Various causes may induce fever symptoms after IHCA. Clinical studies have indicated that infection is responsible for half of fever cases. Additionally, thermoregulatory problems may be involved with fever of unknown origin (14). At the same time, these factors are among the leading causes of death in patients resuscitated from IHCA. Research by Seguin et al. indicated that sepsis and brain trauma are two fundamental causes of poor survival and are independent risk factors for fever (15). However, whether these mortality-related factors influence the outcome by regulating the fever process is still unknown. The main purpose of this research was therefore to determine whether fever has a direct effect on survival or an indirect effect as a mediator. Furthermore, the exact proportion of the indirect effect mediated by fever was investigated, enabling the development of guidelines for the clinical control of fever. To more precisely measure fever, the incidence density of fever (IDF) was adopted. We hypothesized that the IDF acts as a mediator and is associated with a decreased survival rate.

## Methods

### Study Design

A retrospective, observational, single-center study was performed in the tertiary hospital of Shanghai Jiao Tong University Affiliated Sixth People's Hospital (Shanghai PA; 1766 beds) from January 2011 to December 2017. Patient data were collected from the hospital's electronic record database. The Ethics Committee of Shanghai Jiao Tong University Affiliated Sixth People's Hospital (Approval No. 2018-ky-033k) and the Chinese Clinical Trial Registry (Approval No. ChiCTR1800017004) approved this study.

### Patient Selection

**Figure 1:**
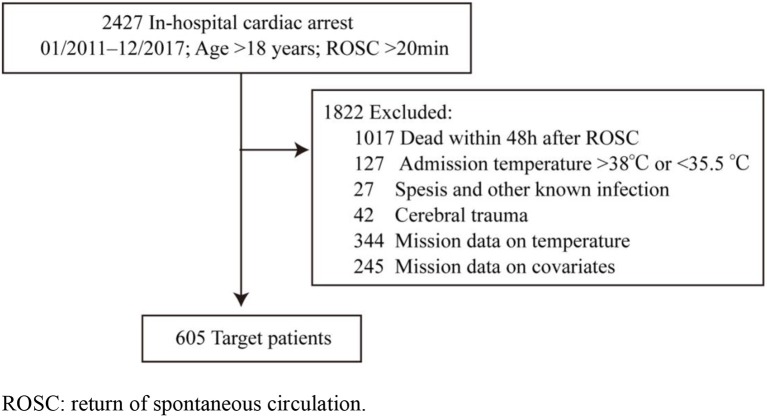
Flow chart for patient inclusion in the study. ROSC, return of spontaneous circulation.

The diagnosis of IHCA was identified according to the *International Classification of Diseases, Ninth Edition, Clinical Modification* (*ICD-10-CM*) code I46.9. For all the patients in the database, the inclusion criteria were as follows: patients older than 18 years; patients who underwent chest compression for at least 2 min during hospital treatment; and patients with the recovery and maintenance of spontaneous circulation > 48 h. The exclusion criteria were as follows: patients with cerebral trauma before admission; patients with a documented do-not-resuscitate order; patients with two or more cardiac arrests; patients with an admission temperature >38°C or <35.5°C; patients with missing temperature records or covariate information; patients with known infection on admission, such as sepsis or pneumonia; patients with antibiotic use before admission; and pregnant patients (Figure 1).

### Data Collection

To ensure validity, the data were reviewed by two trained researchers. The following data were collected from each patient: age, sex, preexisting illness, first monitored rhythm, location of arrest, duration of cardiopulmonary resuscitation (CPR), hospital admission temperature, chest X-ray, highest procalcitonin (PCT) level, and white blood cell count within 48 h following ROSC, sputum cultures obtained within 48 h following ROSC, and vital status at hospital discharge (alive or dead). The length of hospital stay was calculated from the date of ROSC to the date of death or hospital discharge. We reviewed all available temperature data after ROSC from the medical record, and the total duration of a body temperature higher than 38°C and the initial onset of fever were collected.

### Definitions

IHCA was defined as the absence of pulse or hypoperfusion resulting in ventricular fibrillation, pulseless ventricular tachycardia, pulseless electrical activity, or asystole in in-hospital patients who required extra chest compressions. ROSC was defined as no chest compressions for at least 20 min. According to current guidelines, a positive chest X-ray was defined as detectable infiltrate, and a positive sputum culture was defined as the presence of gram-negative bacilli or gram-positive bacteria (16). Severe brain injury was defined as a Glasgow Coma Scale (GCS) score ≤ 8 at 24 h postresuscitation (17). A cardiovascular event was defined as the occurrence of myocardial infarction requiring coronary angiography during hospitalization. The time of day referred to the occurrence of IHCA during work hours (7:00 a.m. to 5:00 p.m. and day of the week). Antibiotic use was defined as the administration of an antibiotic after ROSC. The outcome was survival to hospital discharge. Fever duration was defined as the total duration of an axillary temperature higher than 38°C during hospitalization, converted into days. The IDF for each patient was calculated by dividing the total duration of a body temperature higher than 38°C by the duration of hospitalization (18). No fever was defined as no episodes of temperature ≥ 38.0°C, prolonged fever was defined as fever longer than 5 days (15, 19), and early fever was defined as fever occurring in the first 2 days after CA (4).

### Statistical Analysis

Categorical variables are presented as counts and percentages. For continuous variables, normality was assessed, and non-normally distributed continuous variables are expressed as medians and 25th and 75th percentiles. Variables related to IDF were evaluated by linear regression, and multivariate logistic regression models were used to determine the potential risk factors of fever on survival to hospital discharge. Model 1 adjusted covariates included the following: diabetes mellitus, GCS ≤ 8, CPR interval, epinephrine dosing, and renal replacement therapy. Model 2 adjusted covariates included model 1 plus the potential confounders of procalcitonin, positive chest X-ray, endotracheal intubation, and central venous catheter. Model 3 adjusted covariates included model 2 plus the potential confounders of neuromuscular blockers, corticosteroids, and antibiotic therapy. Model 4 adjusted covariates included model 3 plus the potential confounders of NSAIDs, acetaminophen and physical cooling (**Table 3**). Subsequently, the logistic regression models, which contained other variables that may affect discharge survival, were included in these models. The variables in the logistic regression model are shown in **Table 4**. By using the mediation package in R, we detected whether the relevant variables affected the survival outcome via IDF (20). For each analysis, the null hypothesis was assessed at a two-sided significance level of 0.05, and all analyses were analyzed using PASW/SPSSTM software version 25 (IBM Inc., Chicago, IL, USA) or R version 3.6.2. A probability value of *P* < 0.05 was considered significant.

## Results

### Clinical Characteristics of the Study Population

In this retrospective cohort study, a total of 2,427 patients suffered from IHCA and ROSC after cardiopulmonary resuscitation, of whom 605 were eligible for inclusion in our study. The flow diagram of patient inclusion is shown in Figure 1. The median age of the patients was 69 years old [interquartile range (IQR) 60–80], of whom 48.8% were male (*n* = 297). The median duration of cardiopulmonary resuscitation was 19 min [(IQR) 10–28.5 min]. The location of cardiac arrest was as follows: 45.8% in the general wards, 16% in emergency departments, and 38.2% in intensive care units. Positive chest X-ray was identified in 42% (255/605) of the patients, and positive sputum culture was found in 31.7% (192/605). More than half of the patients had undergone endotracheal intubation, and 67.2% (407/605) and 23.6% (143/605) of the patients had received central venous catheterization. The median IDF was 0 [(IQR) 0–0.42], and the total rate of survival to hospital discharge was 26.9% (164/605) (Table 1). Prolonged fever and short-term fever were noted 16% (97/605) and 28.1% (170/605) of the patients, respectively. Early fever and delayed fever patients were noted in 17.2% (104/605) and 26.9% (163/605) of the patients, respectively. Antipyretic therapy including acetaminophen and non-steroidal anti-inflammatory drugs (NSAIDs) such as ibuprofen, ketoprofen, diclofenac, and physical cooling were indicative of the ice cap. Of these, acetaminophen controlled 32.6% (197/605) and NSAIDs and physical cooling controlled 3.5% (21/605) and 10.6% (64/605) of these cases, respectively.

**Table 1:** Baseline characteristics of the subjects.

**Characteristics**	***n* (%) /Mean SD/median (range)**
Age, years	69 (60–80)
Male (%)	297 (48.8%)
Admission temperature (°C)	36.9 (36.7–37.1)
Hypertension	339 (56%)
Diabetes mellitus	159 (26.2%)
Respiratory diseases	120 (19.7%)
Congestive heart failure	73 (12%)
Hepatic insufficiency	61 (10%)
Renal insufficiency	79 (13%)
Arrest Location (%)	
General ward	277 (45.8%)
Emergency ward	97 (16%)
Intensive care unit	231 (38.2%)
Time of day	317 (52.3%)
Cardiovascular event	140 (23.1%)
VT/VF	78 (12.9%)
CPR time (minutes)	19 (10–28.5)
Epinephrine dosing (mg)	4 (2–7)
GCS ≤ 8	61 (10%)
Blood transfusion	179 (29.5%)
Endotracheal intubation	407 (67.2%)
Central venous catheter	143 (23.6%)
Positive chest X-ray	254 (42%)
Positive sputum culture	192 (31.7%)
Neuromuscular blockers	96 (15.9%)
Corticosteroids	3 (0.5%)
Renal replacement therapy	23 (3.6%)
Acetaminophen	197 (32.6%)
NSAIDs	21 (3.5%)
Physical cooling	64 (10.6%)
Hospital length of stay, days	10 (4–25)
Fever time, days	0 (0–4.5)
Incidence density of fever	0 (0–0.42)
No fever	338 (55.9%)
Short-term fever	170 (28.1%)
Prolonged fever	97 (16%)
No fever	338 (55.9%)
Early fever	104 (17.2%)
Delayed fever	163 (26.9%)
Survival to hospital discharge	164 (26.9%)

*Continuous variables are presented as medians (25–75th percentiles); categorical variables are presented as numbers (percentages). CPR, cardiopulmonary resuscitation; GCS, Glasgow Coma Scale; IHCA, in-hospital cardiac arrest; VT, ventricular tachycardia; VF, ventricular fibrillation; NSAIDs, non-steroidal anti-inflammatory drugs*.

### Factors Associated With the Incidence Density of Fever (IDF)

To determine the risk factors associated with IDF, linear regression was adopted, and basic characteristics, infectious factors, and non-infectious factors were taken into account. Our results showed that positive chest X-ray (Beta = 0.07, 95% CI, 0.02–0.12, *P* = 0.008), central venous catheter (Beta = 0.06, 95% CI, 0.003–0.123, *P* = 0.04), GCS ≤ 8 (Beta = 0.20, 95% CI, 0.12–0.29, *P* **<** 0.001), and IHCA in the emergency ward (Beta = 0.11, 95% CI, 0.04–0.23, *P* = 0.008) were significant factors related to IDF (Table 2).

**Table 2:** Variables related to the incidence density of fever (IDF).

**Characteristics**	**Beta**	**95% C.I**.	***p*-value**
Age (≥ 60)	<0.001	−0.001 to 0.002	0.70
Male	−0.003	−0.06 to 0.05	0.89
Diabetes mellitus	−0.06	−0.12 to 0.002	0.04
VT/VF arrest	0.004	−0.07 to 0.08	0.91
Hepatic insufficiency	−0.05	−0.14 to 0.03	0.24
Renal insufficiency	−0.002	−0.084 to 0.08	0.97
CPR interval (minutes)	0.002	−0.001 to 0.004	0.16
Epinephrine dosing (≥ 4 dose)	0.004	−0.005 to 0.012	0.40
Blood glucose (mmol/L)	0.005	−0.001 to −0.011	0.104
Central venous catheter	0.06	0.003 to 0.123	0.04
Emergency ward	0.108	0.035 to 0.23	0.008
**Infection**			
Positive chest X-ray	0.07	0.02 to 0.12	0.008
Positive sputum culture	0.016	−0.04 to 0.07	0.56
Procalcitonin (ng/ml)	0.004	0.000 to 0.008	0.06
White blood cell count (1,000 cells/L)	0.004	0.00 to 0.009	0.07
**Non-infection**			
GCS ≤ 8	0.20	0.12 to 0.29	<0.001
Blood transfusion	−0.02	−0.07 to 0.04	0.58
Endotracheal intubation	−0.028	−0.113 to −0.058	0.529
Renal replacement therapy	−0.054	−0.186 to 0.079	0.425
Corticosteroid	−0.03	−0.71 to 0.19	0.06
Neuromuscular blocker	−0.18	−0.10 to 0.07	0.67
Antibiotic therapy	−0.03	−0.08 to 0.02	0.28

*Beta coefficients represent the influence of various predictors on model variables. CPR, cardiopulmonary resuscitation; GCS, Glasgow Coma Scale; VT, ventricular tachycardia; VF, ventricular fibrillation*.

### Risk Factors Related to Survival to Hospital Discharge

The impact of fever on the survival of patients discharged from the hospital was analyzed from multiple perspectives. IDF was defined as the fever duration divided by the hospitalization duration, while fever was divided into short-term fever and prolonged fever according to the duration of fever and early fever and delayed fever according to the onset of fever. At the same time, four regression models were established. Model 1 adjusted covariates of patients' basic disease characteristics; model 2 added procalcitonin, positive chest X-ray, endotracheal intubation and central venous catheter as potential infection confounders; model 3 added factors such as neuromuscular blockers, corticosteroids, renal replacement therapy and antibiotic therapy, which may affect body temperature; and model 4 added antipyretic treatments such as NSAIDs, acetaminophen and physical cooling. The results indicated that IDF (OR = 0.36, 95% CI, 0.13–0.97, *P* = 0.04) was an independent risk factor associated with survival to hospital discharge. After adjusting for fever-related confounding factors for prolonged fever, the results remained significant (adjusted OR = 0.13, 95% CI, 0.06–0.29, *P* < 0.001) (Table 3). While short-term fever, early fever and delayed fever did not show a significant impact on patient survival, the results were adjusted OR = 0.94, 95% CI, 0.74–1.38, *P* = 0.34, adjusted OR = 1.28, 95% CI, 0.60–2.72, *P* = 0.52, adjusted OR = 0.82, 95% CI, 0.42–1.60, *P* = 0.56, respectively. Therefore, the results of our study show that the initial onset time of fever and a shorter duration of fever have no significant effect on patient prognosis, while a fever lasting more than 5 days or a higher IDF are negatively associated with hospital discharge.

**Table 3:** Association between fever and survival to hospital discharge with different multivariate logistic regression models.

	**Model 1**		**Model 2**		**Model 3**		**Model 4**	
	**Odds ratio** **(95% CI)**	***P*-Value**	**Odds ratio** **(95% CI)**	***P*-Value**	**Odds ratio** **(95% CI)**	***P*-Value**	**Odds ratio** **(95% CI)**	***P*-Value**
IDF	0.28 (0.14–0.56)	<0.001	0.30 (0.15–0.61)	0.001	0.31 (0.16–0.62)	0.001	0.36 (0.13–0.97)	0.04
No fever	Reference		Reference		Reference		Reference	
Short-term fever	0.66 (0.41–1.06)	0.08	0.64 (0.39–1.06)	0.08	0.64 (0.39–1.06)	0.08	0.94 (0.74–1.38)	0.34
Prolonged fever	0.14 (0.07–0.28)	<0.001	0.14 (0.07–0.27)	<0.001	0.13 (0.07–0.27)	<0.001	0.13 (0.06–0.29)	<0.001
No fever	Reference		Reference		Reference		Reference	
Early fever	0.73 (0.42–1.30)	0.29	0.78 (0.43–1.40)	0.40	0.38 (0.44–1.37)	0.38	1.28 (0.60–2.72)	0.52
Delayed fever	0.43 (0.26–0.70)	0.001	0.45 (0.27–0.74)	0.002	0.50 (0.31–0.81)	0.005	0.82 (0.42–1.60)	0.56

*Model 1 adjusted covariates: diabetes mellitus, GCS ≤ 8, CPR interval, epinephrine dosing, renal replacement therapy. Model 2 included model 1 plus the potential confounders of procalcitonin, positive chest X-ray, endotracheal intubation, central venous catheter. Model 3 included model 2 plus the potential confounders of neuromuscular blockers, corticosteroids, antibiotic therapy. Model 4 included model 3 plus the potential confounders of NSAIDs, acetaminophen, physical cooling. CPR, cardiopulmonary resuscitation; CI, confidence interval; GCS, Glasgow Coma Scale; NSAIDs, non-steroidal anti-inflammatory drugs*.

To further determine the risk factors associated with survival to hospital discharge, we performed univariate and multivariate analyses for the whole cohort. A total of 20 potential risk factors were included (Table 4), and the results revealed that arrest location, epinephrine dose, blood transfusion, endotracheal intubation, central venous catheter, and positive chest X-ray were statistically significant in the univariate logistic regression analysis. Variables associated with survival to hospital discharge with *P* values ≤ 0.2 in the univariate analysis were included in the multivariate logistic regression. In the multivariate logistic regression analyses, endotracheal intubation (adjusted OR = 0.47, 95% CI, 0.33–0.69, *P* < 0.001), central venous catheter placement (adjusted OR = 0.54, 95% CI, 0.34–0.89, *P* = 0.01), and positive chest X-ray (adjusted OR = 0.67, 95% CI, 0.46–0.98, *P* = 0.04) were found to be independent risk factors associated with survival to hospital discharge (Table 4).

**Table 4:** Univariate logistic regression and multivariate logistic regression analyses assessing the impact of factors on survival to hospital discharge.

**Characteristics**	**Unadjusted**			**Adjusted**		
	**OR**	**95% C.I**.	***p*-value**	**OR**	**95% C.I**.	***p*-value**
Age, years	0.992	0.980-1.003	0.155	-		
Male	1.172	0.818-1.681	0.386	-		
Hypertension	0.803	0.555-1.161	0.244	-		
Diabetes mellitus	1.034	0.684-1.562	0.875	-		
Respiratory diseases	1.253	0.799-1.964	0.326	-		
Congestive heart failure	1.431	0.816-2.511	0.211	-		
Hepatic insufficiency	1.383	0.780-2.452	0.268	-		
Renal insufficiency	1.353	0.803-2.279	0.256	-		
Arrest Location						
General ward	Reference					
Emergency ward	0.559	0.342-0.913	0.020	-		
ICU	0.519	0.312-0.863	0.011	-		
Time of day	0.877	0.612-1.255	0.472	-		
VT/VF	1.431	0.816-2.511	0.211	-		
CPR time (minutes)	0.988	0.973-1.003	0.122	-		
Epinephrine dosing (mg)	1.067	1.009-1.130	0.024	1.05	0.99-1.12	0.76
Blood transfusion	0.613	0.404-0.931	0.022	0.69	0.45-1.07	0.09
Endotracheal intubation	0.459	0.317-0.665	0.000	0.47	0.33-0.69	<0.001
Central venous catheter	0.522	0.326-0.834	0.007	0.54	0.34-0.89	0.01
Positive chest X-ray	0.636	0.438-0.924	0.018	0.67	0.46-0.98	0.04

*CPR, cardiopulmonary resuscitation; CI, confidence interval; GCS, Glasgow Coma Scale; VT, ventricular tachycardia; VF, ventricular fibrillation; OR, odds ratio*.

### Mediation Analysis

**Figure 2:**
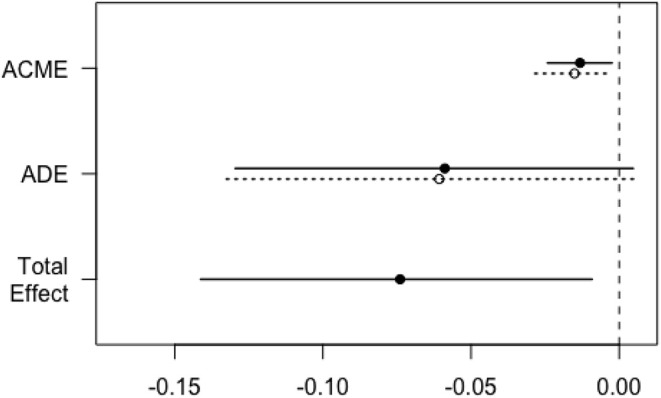
Direct, indirect, and total effect of positive chest X-ray on the survival rate mediated by the IDF. The solid line represents the positive chest X-ray, and the dashed line represents the negative chest X-ray. ACME, average causal mediation effect; ADE, average direct effect; IDF, incidence density of fever.

**Figure 3:**
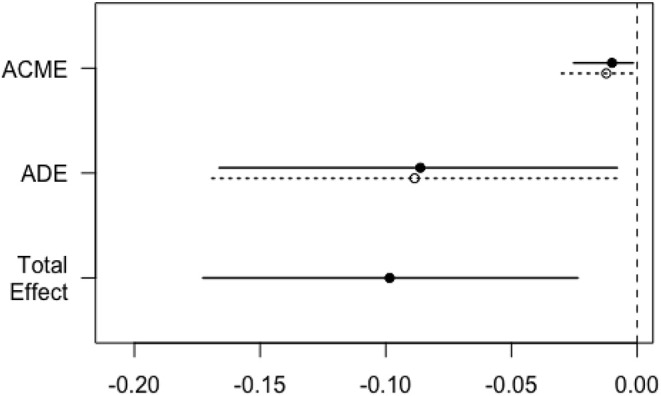
Direct, indirect, and total effects of central venous catheters on the survival rate mediated by the IDF. The solid line represents the patients who underwent central venous catheter, and the dashed line represents the patients without central venous catheter. ACME, average causal mediation effect; ADE, average direct effect; IDF, incidence density of fever.

**Figure 4:**
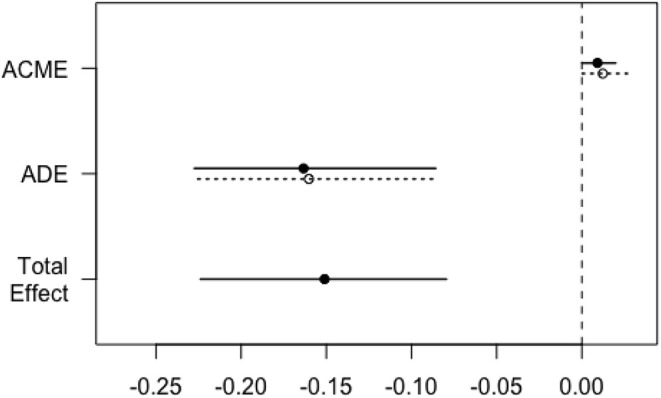
Direct, indirect, and total effects of endotracheal intubation on the survival rate mediated by the IDF. The solid line represents patients who underwent endotracheal intubation, and the dashed line represents patients without endotracheal intubation. ACME, average causal mediation effect; ADE, average direct effect; IDF, incidence density of fever.

To explore the underlying causal mechanism, a causal mediation analysis was performed, and the IDF was designated as the mediator. This analysis divided the total effect into direct effects and indirect effects (20), in which indirect effects represented the causal mechanism through IDF and direct effects represented all other mechanisms. As shown in Table 5, a positive chest X-ray influenced the survival to hospital discharge through the mediating effect of IDF, where the average causal mediation effect (ACME) was −0.01 (95% CI −0.03 to 0.00; *P* < 0.001), the average direct effect (ADE) was −0.06 (95% CI −0.13 to 0.00; *P* = 0.12), and the proportion of the mediated effect was 19% (95% CI 0.02–1.14; *P* = 0.04) (Figure 2). Central venous catheter had an impact on the partially mediated outcome, with an ACME of −0.01 (95% CI −0.03 to 0.00; *P* < 0.001) and an ADE of −0.09 (95% CI −0.16 to −0.01; *P* = 0.04), and the proportion of the mediated effect was 10% (0.01–0.52, *P* = 0.02) (Figure 3). Endotracheal intubation had a non-significant average indirect effect of 0.01 (95% CI 0.00–0.02; *P* = 0.06) (Figure 4).

**Table 5:** Average causal mediation effect through IDF on survival to hospital discharge of positive chest X-ray, central venous catheter, and endotracheal intubation.

**Variables**	**Indirect effect** **(ACME, 95% CI)**	**Direct effect** **(ADE, 95% CI)**	**Total effect** **(95% CI)**	**Proportion mediated** **(95% CI)**
Positive chest X-ray	−0.01 (−0.03 to 0.00)	−0.06 (−0.13 to 0.00)	−0.07(-0.14 to −0.01)	0.19 (0.02 to 1.14), *P =* 0.04
Central venous catheter	−0.01 (−0.03 to 0.00)	−0.09(−0.16 to −0.01)	−0.10(−0.17 to −0.02)	0.10 (0.01 to 0.52), *P =* 0.02
Endotracheal intubation	0.01 (0.00 to 0.02)	−0.16(−0.22 to −0.09)	−0.15(−0.22 to −0.08)	−0.06 (−0.20 to 0.00), *P* = 0.06

*Total effect: the sum of the direct and indirect effects. Proportion mediated: the coefficient of the indirect and total effects. ACME, average causal mediation effect; ADE, average direct effect; IDF, incidence density of fever*.

## Discussion

This study focused on the risk factors related to the rate of survival to hospital discharge of patients with IHCA. We found that the IDF (duration of fever divided by the length of the hospital stay) and prolonged fever (fever duration longer than 5 days) affected the survival rate. In addition, fever-related factors such as central venous catheter placement and positive chest X-ray were found to reduce patient survival to hospital discharge. Through causal mediation analysis, it was shown that IDF mediated the reduction in survival caused by a positive chest X-ray and venous catheter placement.

Previous studies reported that the incidence of fever was ~42% in the first 2 days of resuscitation, and it is related to a poor prognosis (10). The results of our study showed a relatively lower incidence of early fever, which was 17.2%. Meanwhile, after adjusting for antipyretic confounding factors such as antibiotic therapy, NSAIDs, acetaminophen and physical cooling, early onset and delayed onset of fever showed no significant effect on patient prognosis. This could be attributed to the fact that previous studies mainly involved patients with out-of-hospital cardiac arrest, while the main cause of death in such patients is brain injury (4, 10). Non-infective fever with brain injury was more likely to occur within 72 h of admission, and it is associated with a poor outcome (21). Fever of unknown origin after brain injury is called central fever and is clearly indicated by clinical observations; this type of fever can increase the brain metabolic rate and intracranial pressure and aggravate excitotoxicity generation and apoptosis (22, 23), further exacerbating brain injury in a cyclic pattern. By contrast, the subjects of our study were IHCA patients, and these patients underwent bystander-administered life support, which is more rapid and effective in IHCA than in OHCA patients, resulting in a shorter no-flow duration and low-flow duration and, ultimately, in less brain injury (1). Thus, the incidence of early fever was relatively low. However, multiple comorbidities and multiorgan failure are more common in IHCA patients (1, 24). Concerning our results, the relative and absolute duration of fever, which is a relatively higher IDF and fever lasting more than 5 days, was related to a poor survival rate. The mortality rate in the prolonged fever group was 62.5 vs. 29.5% in the shorter-term fever group (19), and higher mortality was associated with longer hospital stays. Prolonged fever may be a representation of certain diseases, such as lung or venous catheter infection, which is shown in our results.

Fever is caused by the upward movement of the central body temperature set point triggered by a pyrogenic source or damage to the central body temperature adjustment point due to brain injury (25, 26). Infection is an independent risk factor for prolonged fever (15). Previous studies found that 52% of fever in critically ill patients is caused by infection, most of which are lower respiratory tract infections and bloodstream infections, especially in patients with diabetes, mechanical ventilation, and renal dialysis. The placement of a venous catheter is the primary reason for bloodstream infections (27). In our retrospective and observational study, fever was found to be related to a positive chest X-ray and central venous catherization but not to endotracheal intubation (Table 2). Endotracheal intubation, as a necessary treatment to ensure oxygenation, reduces the risk of aspirating gastric contents and removes airway secretions in coma patients resuscitated from IHCA; thus, it does not cause fever in patients. Although the clearance of airway secretions may lead to a reduced risk of fever, prolonged endotracheal intubation may lead to pulmonary infection, which is one of the main causes of fever. Endotracheal intubation has been reported to be a prerequisite for the development of ventilator-associated pneumonia, which may manifest as a positive chest X-ray (28). In addition, GCS ≤ 8 after ROSC was also a risk factor associated with fever in our study. This finding is consistent with the results of a previous study reporting that critical neurologic illness patients had a high risk of non-infectious fever, which may be related to the impairment of body temperature regulation centers (29).

In this study, the survival rate of IHCA patients was 26.9%, which agrees with the average survival rate to hospital discharge of 25% reported by the American Heart Guidelines-Resuscitation (30). After adjusting for confounding factors, we found that fever, a positive chest X-ray, central venous catheter placement, and endotracheal intubation were independent risk factors associated with survival outcomes, implying that infection of the lungs or blood may contribute to detrimental survival outcomes partially via the mediating effect of fever. To the best of our knowledge, this study is the first to report the impact of IDF as a mediator on the survival outcomes of IHCA patients. We revealed that a positive chest X-ray had a 19% impact on reducing the survival rate through the mediating effect of IDF and that IDF mediates the impact of central venous catheter placement. Park (31) reported that fever contributed to pronged mechanical ventilation for critically ill patients. Moreover, prolonged endotracheal intubation may increase the risk of ventilator-associated pneumonia, indicated as a positive chest X-ray (32). Furthermore, pneumonia is a significant predictor of poor outcome among patients resuscitated from IHCA (33). Studies have shown that the placement of a central venous catheter increases the risk of blood-borne complications and increases mortality, and the early removal of a central venous catheter can help reduce mortality in certain subgroups (34). With regard to critically ill patients with cardiac arrest, a central venous catheter is both a necessary measure and a risk factor that may decrease survival. Half of the cases of fever in critically ill patients are due to infectious etiologies. Our results suggest that fever has an indirect impact on the reduction in survival induced by lung disease and central venous catheter placement, which indicates that controlling fever is crucially important for improving the IHCA survival rate. However, inflammation-associated fever is commonly regarded as a way to enhance the host response (35). It remains unclear through what mechanism fever mediates the detrimental effects of positive X-rays and central venous catheter placement.

There are several limitations to this study that should be noted. First, this study was a retrospective, observational, single-center study that inevitably had inherent bias. In addition, some data were missing due to various reasons, which may have resulted in underrepresented results. Second, considering that heat transfer-related factors can affect the axillary temperature data, interventions that may influence fever were used to adjust the multivariate regression models. Third, despite our best efforts, there may have been unmeasured confounding factors that were not considered in our multivariate analyses. The results of this study require further confirmation in future work.

## Conclusion

In our cohort of IHCA patients, a higher IDF and prolonged fever were negatively related to survival outcomes. Additionally, a positive X-ray and central venous catheter placement also reduced the survival rate of patients, affecting this outcome through the mediating effect of IDF. These results emphasize the need for the prevention and treatment of prolonged fever, which could improve the survival rate of IHCA patients.

## Data Availability Statement

Datasets are available on request: The raw data supporting the conclusions of this article will be made available by the authors, without undue reservation, to any qualified researcher.

## Ethics Statement

The studies involving human participants were reviewed and approved by the ethics committee of Shanghai Jiao Tong University Affiliated Sixth People's Hospital (Approval No. 2018-ky-033k) and the Chinese Clinical Trial Registry (Approval No. ChiCTR1800017004) approved this study. Written informed consent for participation was not required for this study in accordance with the national legislation and the institutional requirements.

## Author Contributions

Experiments were designed by DC and were performed by YG, YH, YL, and XW. The manuscript was written by YH and DS and edited by DC and YG.

### Conflict of Interest

The authors declare that the research was conducted in the absence of any commercial or financial relationships that could be construed as a potential conflict of interest.

## Abbreviations

ACME: average causal mediation effect
ADE: average direct effect
IDF: incidence density of fever
CPR: cardiopulmonary resuscitation
GCS: Glasgow Coma Scale
IHCA: in-hospital cardiac arrest
VT: ventricular tachycardia
VF: ventricular fibrillation.
